# Exercise and Cardiac Preconditioning Against Ischemia Reperfusion Injury

**DOI:** 10.2174/1573403X113099990033

**Published:** 2013-08

**Authors:** John C Quindry, Karyn L Hamilton

**Affiliations:** 1 Cardioprotection Laboratory, Department of Kinesiology, Auburn University, Auburn, AL 36849, USA;; 2 Department of Health and Exercise Science, Colorado State University, Fort Collins, CO 80523, USA

**Keywords:** Cardioprotection, myocardial infarction, oxidative stress, physical activity.

## Abstract

Cardiovascular disease (CVD), including ischemia reperfusion (IR) injury, remains a major cause of morbidity and mortality in industrialized nations. Ongoing research is aimed at uncovering therapeutic interventions against IR injury. Regular exercise participation is recognized as an important lifestyle intervention in the prevention and treatment of CVD and IR injury. More recent understanding reveals that moderate intensity aerobic exercise is also an important experimental model for understanding the cellular mechanisms of cardioprotection against IR injury. An important discovery in this regard was the observation that one-to-several days of exercise will attenuate IR injury. This phenomenon has been observed in young and old hearts of both sexes. Due to the short time course of exercise induced protection, IR injury prevention must be mediated by acute biochemical alterations within the myocardium. Research over the last decade reveals that redundant mechanisms account for exercise induced cardioprotection against IR. While much is now known about exercise preconditioning against IR injury, many questions remain. Perhaps most pressing, is what mechanisms mediate cardioprotection in aged hearts and what sex-dependent differences exist. Given that that exercise preconditioning is a polygenic effect, it is likely that multiple mediators of exercise induced cardioprotection have yet to be uncovered. Also unknown, is whether post translational modifications due to exercise are responsible for IR injury prevention. This review will provide an overview the major mechanisms of IR injury and exercise preconditioning. The discussion highlights many promising avenues for further research and describes how exercise preconditioning may continue to be an important scientific paradigm in the translation of cardioprotection research to the clinic.

## INTRODUCTION

In industrialized countries, cardiovascular disease (CVD) is the leading cause of death. Of the major forms of CVD, ischemic heart disease is the most prevalent totaling more than 1 million deaths in the United States annually. In addition to the human toll, CVD is also the most costly diagnostic classification accounting for an estimated $286 billion (15% of total health expenditures) in 2007 U.S. dollars [1]. Ischemic heart disease results in complications including ventricular arrhythmias and congestive heart failure. Scientific and medical advances over recent decades reveal that ischemic pathology encompasses injurious events experienced during both ischemia and reperfusion, collectively described as ischemia-reperfusion (IR) injury [2, 3]. This review will focus on the damage caused by IR injury.

While much is currently known about the cellular events that underpin IR injury, pragmatic solutions remain elusive [4]. A typical PubMed search of peer reviewed manuscripts related to myocardial IR injury yields approximately 14,000 hits, though viable clinical translations are not among the findings. This overview briefly addresses myocardial injury and summarizes empirical evidence which indicates that endurance exercise stimulates a robustly cardioprotective phenotype. Exercise induced cardioprotection is classified into four broad categories including 1) tissue and coronary artery remodeling, 2) CVD risk factor modification, 3) post event cardiac rehabilitation, and 4) cardiac preconditioning. Each facet of exercise induced cardioprotection is discussed, though the focus of this review is exercise preconditioning against IR injury. Specific attention is given to understanding foundational research which describes the exercise preconditioned phenotype. The putative mechanisms responsible for myocardial protection are also discussed with particular emphasis placed on the role of endogenous antioxidant defenses. Given recent exercise preconditioning research findings, this review highlights unique facets of the exercise stimulus as a valuable experimental approach in the exploration of future therapeutics against IR injury.

## MECHANISMS OF ISCHEMIA REPERFUSION INJURY

### Fundamentals of Myocardial Injury

Clinical manifestations of coronary artery disease complicate the continual necessity of ventricular contractile function. Given the very tight regulation of cardiac bioenergetics, oxygen deprived myocardial tissue downstream of a partially or fully obstructed coronary artery quickly exhibits signs of energy supply-demand mismatch. Untoward clinical outcomes are evident within seconds to minutes following a coronary occlusion and reflect cellular dyshomeostasis [5]. Restoration of blood flow, while necessary, is ultimately more deleterious than the proceeding ischemia due to a concert of pathological mechanisms [6]. The magnitude of injury produced during ischemic and reperfusion periods generally accrues in proportion to ischemic duration [3, 6, 7], with myocardial tissue death observed after approximately 20 minutes of unremitting ischemia [2]. This time threshold is thought to be somewhat fluid based on many factors including ventricular mass involvement, metabolic state of the cardiac tissue, and pre-ischemic tissue health [8, 9]. Recent findings further reveal that necrotic, apoptotic, and autophagy cellular processes are responsible for cardiomyocyte loss during extended duration IR [10-13]. 

The ischemic-reperfused myocardium, however, is characterized by an evolving pathology marked by clinically relevant benchmarks prior to tissue death. Ventricular arrhythmias appear within 1-5 minutes following the onset of myocardial ischemia. While ventricular pump function may exhibit little or no contractile deficit in the early moments of an ischemic event, the onset of cardiac arrhythmias are potentially lethal. Widespread use of automated external defibrillators as a lifesaving device underscores ventricular arrhythmia lethality. Myocardial stunning, marked by a measurable loss in contractile function in the absence of tissue death, occurs as ischemia duration exceeds 5 minutes. In cases of timely reperfusion, contractile deficits due to myocardial stunning are transient based upon observations of restored ventricular pump function within days to weeks [14, 15]. Notably, the three major levels of IR injury described are cumulative in that arrhythmias and contractile deficits persist as ischemic duration extends to the point of myocardial stunning and tissue death [3, 7, 10, 14-16].

Decades of reductionist science have revealed many of the cellular events, responsible in mass, for the clinical manifestations of IR injury described above. These cellular mechanisms are an interconnected cascade of events which occur in the metabolic aftermath of IR. While IR injury is complex by virtue of the host of pathological mediators identified to date, the various mechanisms can be broadly grouped into two parent categories related to either oxidative stress or calcium overload. As discussed shortly, independent oxidative stress and calcium overload mechanisms promote IR injury in concert. The reader is directed toward several excellent reviews which further detail the mechanisms of IR injury [3, 7, 10, 14-16].

### Oxidative Stress

Oxidative stress is a cornerstone of IR injury [17]. A fundamental understanding of free radicals and their production is required. Free radicals are very reactive molecules due to the fact that they contain unpaired electrons in their outer orbital. As such, free radicals act as oxidizing agents in oxidation-reduction (redox) reactions within biological systems. Oxygen-based nonradical derivatives also participate in related oxidation reactions. Free radicals and redox reactive nonradicals are collectively called “reactive oxygen species” (ROS) and are the initiating reactants of oxidative stress chain reactions. The term oxidative stress is a generic term which describes either ROS-generated damage to cellular constituents or an overproduction of oxidants. Oxidant overproduction is thought to be a relative phenomenon compared to the radical quenching capacity of available antioxidants. Modern understanding now holds that oxidative stress represents a chronic imbalance which results in detrimental outcomes in cellular redox signaling and control systems [18]. This current understanding of oxidative stress reconciles that ROS are as essential to normal cellular signaling and health as they are contributors to disease pathologies. 

During myocardial IR, ROS production increases dramatically and is associated with all three levels of injury [15]. The major sources of ROS in ischemic myocardia include respiring mitochondria, cellular oxidases, and nitric oxide producing synthases. Nitrogen centered radicals dismutate to form other reactive species. While nitrogen and oxygen centered ROS are short lived as a function of their reactive nature, some forms diffuse through cell and organelle membranes, thereby spreading oxidative stress within and between cells. Within the mitochondria, complex I and III of the electron transport chain are major sites of ROS formation [9]. While these ROS dismutate spontaneously or through interaction with endogenous antioxidant defenses they also damage the mitochondria, further impacting myocardial energy deficits. This fact is particularly important in instances of intermittent IR in those with CVD. Additional ROS sources include autoxidation of catecholamines, phagocytic burst by white blood cells, and reactions with disrupted transition metals including iron and copper [19]. Xanthine oxidase and NADPH/NADH oxidases are potent cytosolic ROS producing enzymes within ischemic myocardia. Enzymatic ROS production is exacerbated by cytosolic calcium (Ca^2+^) elevation and indicate that oxidative stress and calcium dysregulation act synergistically to promote IR injury. Inflammatory cells brought to the ischemia region during and following ischemia exert further oxidative stress. In addition to respiratory burst by phagocytes, inflammatory ROS production is promoted by the enzymes NADPH oxidases, cyclooxygenase-2 (COX-2), and inducible nitric oxide synthase (iNOS) [14].

Both ischemia and reperfusion are marked by oxidative stress, though most damage is experienced during reperfusion [3]. Cellular damage due to oxidative stress impacts nearly all cellular constituents including protein, lipids, and nucleic acids. Polyunsaturated lipids within organelle and cellular membranes are oxidized by the process of lipid peroxidation. Oxidation of membrane fatty acids prevents normal cell function due to decrements in membrane fluidity and function. Protein function and structure are compromised by oxidation of specific amino acids with exposed thiol groups that readily participate in redox reactions. The ensuing damage results in formation of carbonyl side chains on oxidized amino acids which can be quantified biochemically [20]. The impact of protein carbonyl formation during IR is vast and includes decrements in enzyme function, receptor activity, structural integrity, damage to contractile filaments, and mechanisms of cellular transport [21]. Similarly, DNA bases are also susceptible to oxidative damage resulting in genomic mutations which impacting cell health [15, 19]. The collective effect is that oxidative stress is an undeniable outcome during IR. Few facets of cellular function and integrity are spared from oxidative damage as endogenous antioxidant defenses are overwhelmed by ROS production during extended duration IR.

### Calcium Dysregulation

Calcium concentrations are tightly regulated within the cytosol and mitochondria of fully perfused myocardial tissue. As with other major physiologic ions in the heart, Ca^2+^ regulation is bioenergetically costly. During IR, Ca^2+^ dysregulation rapidly rises to pathologic levels as ATP production declines [9]. Similar to oxidative stress, Ca^2+^ dysregulation is observed during both ischemia and reperfusion [22, 23]. Hydrogen ions accumulate concomitant to Ca^2+^ as glycolytic metabolism becomes the dominant metabolic pathway during ischemia. Sarcolemmal Na^+^-H^+^ exchanger activity increases dramatically to counter the resulting decrease in cellular pH. This response partially reconciles the cellular Ca^2+ ^load by compromising the normally low cellular Na^+^ levels. Rising myocardial Na^+^ concentrations undermine the Na^+^-K^+^ currents required to drive myocardial excitability during each cardiac cycle. The collective ionic disturbance is further compounded by the fact that Na^+^-K^+^ ATPase activity is diminished severely in the absence of abundant ATP. Activity rates of Na^+^-Ca^2+^ antiporters are increased in an effort to preserve cellular membrane potential, exacerbating cellular Ca^2+^ overload [14, 23].

Interconnectedness between Ca^2+^ overload and oxidative stress includes the fact that membrane permeability is increased significantly by ROS. Calcium overload also impacts cell health through activation of calpain proteases [24]. Proteolytic cleavage by calpains is not limited to the ‘cellular machinery’ associated with normal cardiac function but also triggers mediators of cellular apoptosis [25]. In combination with inflammation, Ca^2+^ overload is responsible for damage caused to cardiac tissue during both ischemia and reperfusion. 

### Exercise Induced Cardioprotection

Exercise induced cardioprotection is well established concept. A classic study by Paffenbarger demonstrated that in contrast to formal exercise, work related physical activity alone was associated with lower incidence of CVD mortality. Concluding this early work, Paffenbarger *et al.*, state “*the association between work activity and coronary mortality…suggests that physical activity influences the myocardium or its function more than the atherosclerotic process*” [26]. This notion that the myocardium possesses endogenous disease resistance characteristics is remarkable in that it long predates mechanistic understanding of IR injury and exercise preconditioning. Empirical evidence supporting exercise preconditioning against IR injury includes highly controlled experimental investigations using a variety of animal models of exercise and IR injury. Important to biomedical applicability in humans, virtually all of these studies examine clinically relevant cardiac outcomes and gene products common to mammalian physiology. 

### The Many Facets of Exercise Induced Cardioprotection

The term exercise induced cardioprotection covers a broad series of observations. CVD incidence is improved by risk factor modification in those who exercise. Alteration in blood lipids [27], blood glucose control [28], hypertension [29], and obesity [30] are improved by regular exercise participation. Risk factor modifications due to exercise provide independent and synergistic improvements in CVD risk [1, 31, 32]. Hemodynamic demands of regular exercise exposure elicit architectural remodeling of both the coronary circulation [33] and ventricle musculature [34, 35]. Exercise-driven outcomes collectively diminish the likelihood and severity of ischemic events. Given the millions of IR events annually [1], rehabilitative exercise following IR injury is also a well-accepted therapeutic intervention in those with a variety of cardiac diseases [36-38]. 

Exercise preconditioning against IR injury is the least understood facet of exercise induced cardioprotection. By definition, exercise preconditioning is a cardiac phenotype that is resistant to IR injury subsequent to the therapeutic condition of exercise. Recent experimental evidence now demonstrates that IR injury resistance conferred by exercise is independent of muscular and blood flow alterations and is attributed to myocardial-specific biochemical adaptations derived by exercise. Research conducted over the last decade reveals that exercise preconditioning is a robust polygenic response. Current understanding of exercise preconditioning against IR injury is overviewed in the subsequent sections. 

### Observational Evidence of Exercise Preconditioning

Empirical research demonstrates that the exercised heart is resistant to IR injury including arrhythmia [39, 40], myocardial stunning [41-47], and myocardial infarction [48-52]. Distinction between exercise mediated infarct protection due to necrotic and apoptotic tissue death now exists [52-54]. Recent work reveals for the first time that anti-apoptotic effects post IR may include preservation of basal autophagy levels in the hours acutely following an IR event [55]. Further supporting the robust stimulus as a countermeasure against IR injury, exercise induced preconditioning occurs independent of sex [43, 45, 52, 53, 56, 57] and age [44, 54, 58]. 

### Exercise Dose and Cardiac Preconditioning

The exercise dose required to elicit myocardial protection is relatively low. Despite the fact that this line of research is derived exclusively from animal experiments, the exercise intensities are proportional to an approximation of American College of Sports Medicine (ACSM) guidelines for health and fitness. An early investigation demonstrated preservation of ventricular pressure development during IR in rats exposed to 10 weeks of treadmill exercise [35] at an intensity of ~75% VO_2_max [59, 60]. Several landmark studies later demonstrated that short term exercise regimen of 3 and 5 days of similar intensity treadmill exercise preserved ventricular contractility as effectively as 10 weeks of exercise training [35, 49]. Subsequent study revealed that a single 30 minute bout of treadmill running partially prevented necrotic tissue death during IR [61]. The exercise intensity required to achieve IR injury resistance is also relatively low. Rats exposed to 3 days of treadmill exercise at 55% and 75% V O_2_max were equally protected against contractile deficits elicited by IR [46]. Follow-up work using a 3 day exercise regimen further demonstrated preservation of ventricular contractility 9 days following the last exercise session [45]. Collectively, these findings demonstrate that transient exercise preconditioning occurs acutely following exercise and is stimulated by relatively low exercise intensities.

### Putative Mechanisms of Exercise Preconditioning

Research over the last 20 years clearly demonstrates that exercised hearts exhibit significant protection against IR insults, though the protective mechanisms have only just begun to be understood. Given the robust protection observed with just 3 days of moderate intensity exercise, logic would hold that exercise mediated protection would be due largely to biochemical alterations in the exercised myocardium. Understanding that IR injury is mediated primarily by Ca^2+^ dysregulation and oxidative stress subsequent to ATP supply-demand mismatch, exercise preconditioning is likely to stimulate mechanisms which counter these pathological events. Empirical evidence over the last decade reveals that exercise preconditioning is a polygenic response which parallels the major facets of IR injury. Among the potential exercise preconditioning mediators are including heat shock proteins (HSP), Ca^2+ ^handling proteins, ATP-sensitive potassium channels (K_ATP_ channels), and endogenous antioxidants. The following sections overview findings from these studies. 

### Heat Shock Proteins 

A single bout of hyperthermia elicits up-regulation in HSPs within cardiac muscle [62]. Given the thermogenic nature, exercise is among the many physiologic stimuli of HSP up-regulation in the myocardium [39, 47, 50-52, 63-65]. Independent of exercise, HSP overexpression is cardioprotective. While various HSPs mediate cytoprotection, HSP72, an HSP sub-family distinguished by molecular weight, is notably protective against IR injury [62]. 

Investigations of HSPs, including HSP72, as potential mediators of exercise preconditioning have been equivocal [39, 47, 50-52, 62-67]. Early studies repeated across independent labs demonstrated that exercise was associated with both HSP72 overexpression and IR injury prevention [50, 63, 65, 67], however, it remained undetermined whether these observations were epiphenomena. To approach this question, researchers simultaneously countered exercise induced thermogenesis and HSP72 overexpression by exercising animals in a cold environment (4°C) [50]. As compared to animals exercised at room temperature (25°C), cold exposure prevented HSP72 increases in exercised myocardia. When exercised animals from cold and room temperature groups were exposed to experimental IR, it was found that myocardial HSP72 expression was independent of exercise-mediated protection against IR induced ventricular arrhythmias [39] and tissue death [52]. These findings suggest that HSP72 is not essential for exercise preconditioning against IR injury. 

The term ‘heat shock’, however, is a misnomer of sorts in that overexpression can be elicited by oxidative stress and others stimuli imposed by exercise. To examine the contribution of exercise generated oxidants on HSP72 content and cardioprotection, animals were treated with and without an antioxidant cocktail prior to surgically induced IR [51]. Similar to the temperature control studies described previously [50], Hamilton *et al*., observed an attenuation in exercise induced HSP72 up-regulation in antioxidant treated animals [51]. Despite the absence of HSP72 overexpression, animals exhibited similar levels of cardioprotection against IR-induced tissue death as compared to exercised control animals [51]. These findings support previous studies [65] which indicate that HSP72 is not essential for exercise preconditioning against IR injury. While this conclusion is somewhat counterintuitive given the consistent observation of HSP72 up-regulation by exercise, it highlights the fact that exercise elicits redundant mechanisms of cardioprotection. 

### Calcium Handling

Given the role of Ca^2+^ overload in IR injury [41], one would expect exercise preconditioning to bolster mechanisms of Ca^2+ ^handling. Sparse but notable data exist to support exercise mediated improvements in Ca^2+ ^handling. For instance, prevention of myocardial stunning during IR is associated with improved Ca^2+^ handling in exercised hearts [68]. Further indicative of improved Ca^2+ ^control, IR induced activation of the Ca^2+^ sensitive protease, calpain, is significantly attenuated in exercised hearts [69]. In exercised hearts exposed to IR sarcoplasmic/endoplasmic Ca^2+^ ATPase protein content, a common target of IR damage, is preserved [69]. A recent association was also observed in unstressed hearts between prior exercise and improved Ca^2+ ^release to trigger ventricular contractions and minimized Ca^2+ ^leak during ventricular relaxation [70]. This latter observation would presumably translate to attenuated arrhythmia generation and supports the anti-arrhythmic effects described previously in exercised hearts exposed to IR [39, 40]. While further study is required for mechanistic verification of these observations, foundational evidence indicates that exercise induced cardioprotection includes improved Ca^2+^ handling during IR challenges. 

### ATP-sensitive Potassium Channels 

ATP-sensitive potassium channels at the myocardial sarcolemma (SK_ATP_) and inner mitochondrial membrane (MK_ATP_) are important to normal cardiac function and mediate cardioprotection against IR injury with non-exercise stimuli [71, 72]. As the name implies, these energy sensing ion channels are inhibited in the presence of abundant cellular ATP, but open during bioenergetic challenges including IR. Opening of either SK_ATP_ or MK_ATP_ channels prior to IR exert significant cardioprotection against various forms of IR injury. Cardioprotection derived from K_ATP_ channel opening remains incompletely understood, though the SK_ATP_ appears to trigger MK_ATP_ channel activation in some experimental settings [71]. Tentative explanations include shortening of the cardiac cycle, prevention of cytosolic and mitochondrial Ca^2+^ overload, improving bioenergetic availability, and conversely decreasing bioenergetic demand within the ischemic myocardium [73, 74]. Of importance to exercise applications, K_ATP_ channels also open independent of falling ATP levels [73]. This point is important to exercise scenarios in that ATP concentrations are well maintained in the fully perfused myocardium of exercised animals. In the absence of an acute drop in cellular ATP levels of healthy hearts, other mechanisms must trigger K_ATP_ channel opening in response to exercise. Of the known K_ATP_ channel activators, PKCε is a likely candidate in that cytosolic levels of this kinase are elevated in hearts from exercised animals. In non-exercise experiments, PKCε also mediates cardioprotection via K_ATP_ channel opening [75, 76]. In support of this rationale, use of a pharmacologic PKC inhibitor blunted exercise induced cardioprotection. The agonist used in this study, however, was a non-selective PKC inhibitor [77]. Additional research is needed to confirm whether PKCε and K_ATP_ channel activation are mechanistic partners in the prevention of IR injury.

The role of K_ATP_ channels in the exercise preconditioning is only partially understood [40, 57, 78]. In exercised hearts K_ATP_ channel sub-types appear to precondition differently based on sex and the type of IR injury. Based on initial studies in exercised animals, the MK_ATP_ channel mediates protection against IR induced arrhythmias [40] while the SK_ATP_ channel mediates protection against necrotic tissue death [56, 57, 78, 79]. Recent investigation reveals that SK_ATP_ channel mediated protection against tissue death is limited to necrosis and does not include prevention of apoptotic processes [79]. Examination of sex-dependent differences reveal infarct sparing effects are evident in exercised female hearts [56, 57, 78] but not in hearts from exercise males [56, 57, 78, 79]. Nonetheless, aforementioned protection against IR induced ventricular arrhythmias by the MK_ATP_ channel was observed in male rats [40]. As such, additional research is needed to better understand the role of both K_ATP_ channel subtypes across genders and forms of IR injury. 

Future research on the contribution of K_ATP_ channels to exercise induced cardioprotection should consider the feasibility of novel reductionist approaches. The aforementioned research studies relied exclusively upon pharmacologic inhibitors purported to be selective for either the MK_ATP_ or SK_ATP_ channel channels [40, 56, 57, 78, 79]. However, these drugs are criticized for possessing non-specific pharmacologic effects that include potential alterations in myocardial metabolic rates which may confound outcomes of IR investigations [57, 73, 80, 81]. While molecular biology approaches would seem the obvious alternative to pharmacologic investigations, significant limitations remain. K_ATP_ channel knockout models, for instance, are intolerant to exercise making them unsuitable to exercise training applications [82]. Use of antisense oligonucleotides and siRNA technology, while normally well suited to prevent exercise-induced protein overexpression, are also inappropriate in that allosteric activation of constitutive K_ATP_ channels will elicit a cardioprotected phenotype independent of an exercise induced increase in ion channel number [82]. The search for a sustainable means of K_ATP_ channel activation as a countermeasure against IR injury has been ongoing for more than a decade. To date, exercise-based research appears to be among the most promising experimental approaches for uncovering therapeutically viable solutions to K_ATP_ channel activation and cardioprotection. Nonetheless, it is clear that several methodological hurdles must be negotiated to better understand the exact role of K_ATP_ channels in exercise preconditioning against IR injury. 

### Enzymatic and Non-enzymatic Antioxidants

The heart, like other tissues, has well conserved antioxidant defenses to counter oxidant production. While modest fluctuations in redox balance are a normal fact of biology, overwhelming shifts in oxidant production are a corner stone of IR injury [15, 18]. Endogenous antioxidant defenses are broadly categorized as either enzymatic or non-enzymatic antioxidants. These antioxidant defenses protect through various biochemical approaches including catalytic quenching of ROS, protein complexes that minimize ROS interactions, protein chaperones which prevent oxidant damage, and compounds that directly scavenge ROS. Catalytic ROS removal in muscle, including cardiac, is largely accomplished by a networked series of enzymes which include superoxide dismutase (SOD), catalase (CAT), glutathione peroxidase (GPx), peroxiredoxins, and thioredoxins. Two forms of SOD are present within the myocardium, copper zinc SOD (CuZnSOD) in the cytosol and manganese SOD (MnSOD) in mitochondria. In regard to non-enzymatic defense systems, transition metals, especially iron, encased in structural proteins react readily with ROS thereby minimizing oxidant damage. Examples include transferrins, haptoglobins, and metallothionein. HSPs, including HSP72 discussed previously, are among the most important chaperone proteins which possess direct and indirect antioxidant properties. Finally, direct ROS scavenging occurs endogenously by small molecular weight compounds including glutathione, uric acid, and bilirubin. Additional antioxidants are provided through the diet and include vitamin C, α-lipoic acid, and α-tocopherol [83]. 

Of the four broad antioxidant defense categories described above, the enzymatic antioxidant defenses appear to be the most important to exercise induced cardioprotection. Transcriptional regulation of these endogenous antioxidant proteins in response to exercise is controlled largely by non-pathologic oxidants produced during exercise [84]. Recent understanding of this response to exercise reveals an important role of oxidants in transcriptional regulation of ‘phase 2 enzymes’ via activation of the transcription factor Nuclear factor-erythroid-2-related factor 2 (Nrf2) [85]. A simplistic via of Nrf2 activation in response to exercise begins with Nrf2 held inactive by binding to kelch-like ECH-associated protein 1 (Keap1) in the cytosol. Keap1 serves as an oxidant sensor with cysteine residues which are modified in response to redox perturbations that occur during exercise, disturbing the normal ubiquitination and degradation of Nrf2. Stabilized Nrf2 is then phosphorylated potentially by a variety of kinases including mitogen activated protein kinases, PKC, and PI3K/Akt [86, 87]. Activated Nrf2 is then imported to the nucleus to regulate expression of genes containing electrophile-responsive elements (EpRE) also known as antioxidant responsive elements (ARE) [88]. The EpRE is present in promoters of genes for many proteins exerting direct and indirect antioxidant activity, molecular chaperones, and DNA and protein repair and removal as reviewed elsewhere [87]. This antioxidant response is fundamental to the observed exercise induced bolstering of endogenous antioxidant defenses [89, 90]. Indeed this is underscored by a very recent report that acute exercise results in Nrf2 activation and enhanced cardiac antioxidant defenses in wild type mice, with significant blunting of this response and increased susceptibility to myocardial oxidative stress in Nrf2 -/- mice [91].

Of the endogenous antioxidant enzymes described above, and reported to increase with exercise training, MnSOD is the only example with repeated evidence to indicate an essential role in exercise preconditioning against IR injury. MnSOD protein content and activity is consistently elevated in the exercised myocardium while CuZnSOD is not [35, 39, 40, 45-47, 49-52, 61, 92]. A series of experiments by two lab groups employed antisense oligonucleotide gene silencing technology to test the essentiality of MnSOD as a mediator of exercise preconditioning. Antisense oligonucleotides directed against MnSOD blunt exercise induced increases in MnSOD. This approach is scientifically advantageous because alternative approaches including unconditional knockout of MnSOD is lethal in neonates [93]. The first report using this approach demonstrated that exercise provides biphasic protection against myocardial infarction via increases in MnSOD [61]. Subsequent investigations found that MnSOD also prevented IR-induced ventricular arrhythmias [39] but was not essential for preservation of muscle pump function observed in exercised hearts [47]. Follow-up investigation by the same group demonstrated that MnSOD was partially responsible for preventing apoptotic tissue death following IR. These latter findings were associated with preservation of handling Ca^2+^ proteins and prevention of calpain activation [53]. Additional research is needed to understand whether other phase 2 antioxidant enzymes are responsible for exercise mediated protection against IR injury.

### Exercise: A Unique Medicine for Cardiac Preconditioning

Recent research reveals that exercise preconditioning is a unique stimulus for cardioprotection as compared to other major investigative approaches to IR injury protection. Complimentary to exercise-based research, other well-studied forms of cardiac preconditioning include pharmacologic and ischemic preconditioning (IPC). The former approach involves pre-emptive use of pharmacologic agents to induce IR injury resistance [94]. IPC, on the other hand, is a purely experimental paradigm for cardioprotection originating from a now famous study by Murry *et al.*. In this study, short intervals (minutes) of surgical IR in anesthetized dogs produced significant cardioprotection against more clinically relevant IR challenges of extended duration [95]. From a broader scientific perspective, exercise, pharmacologic, and IPC stimuli provide a similar magnitude of IR injury protection [4, 94, 96]. For many years it was assumed that these stimuli were, more or less, interchangeable “hormetic” stimuli [4, 97]. Hormesis is a theory which states that when toxins and other stimuli are present in low, sub-pathologic doses, they will promote good health adaptations [98, 99]. Further consideration of existing data, however, suggests that cardioprotection afforded by exercise, pharmacologic, and IPC stimuli are fundamentally different.

Descriptive and mechanistic evidence support the position that exercise is a unique stimulus for cardiac preconditioning. This new revelation is a departure from past understanding based only on the observation that exercise and ischemic stimuli produce roughly equal biphasic protective responses against experimental IR [14, 61, 97]. The first phase of protection occurs in the minutes to hours following exercise or IPC, and disappears just as quickly. The second phase, while also transient, is a much longer window of IR injury protection and lasts for several days [14, 61, 97]. Across the sub-areas of cardiac preconditioning research, this more robust ”late phase” window of protection is not extensively in the hopes of discovering translatable mechanisms of IR injury prevention. Further examination of late phase protection in exercise and IPC stimulated hearts, however, reveals vastly different protective profiles. IPC mediated protection is lost after a maximum of 96 hours, while exercise mediated protection is maintained for at least 9 days [4, 45]. Time course differences in protection due to exercise and IPC, while purely descriptive in nature, suggest different biochemical mediators are at work in the two experimental models. Further supporting the notion that exercise is a more robust cardioprotective stimulus, we and others have demonstrate that short-term exercise training protects aged hearts [44, 54, 58]. While few IR investigations have been undertaken in aged animals, existing data indicate that IPC and pharmacologic preconditioning are ineffective in eliciting cardioprotection in senescent hearts [100].

Side-by-side comparison of exercise and IPC research studies performed over the last decade indicate that some unique mechanisms mediate preconditioning by IPC and exercise Table **1**. To summarize a wealth of research, cardioprotection following IPC is essentially dependent upon increased activity and protein content of cyclooxygenase-2 (COX-2) and the inducible form of nitric oxide synthase (iNOS) within the myocardium [101, 102]. We investigated whether iNOS and COX-2 were essential mediators of exercise induced cardioprotection. Neither iNOS nor COX-2 levels were over-expressed in young hearts from exercised animals that exhibited cardioprotection against global IR [54, 58]. Given the obvious link between advancing age and heart disease incidence, cardiac iNOS levels increase with advancing age, though a few weeks of exercise training effectively reverses this effect [103]. Building on this understanding, we also demonstrated that myocardial iNOS and COX-2 levels were not associated with anti-apoptotic effects in aged hearts exposed to IR [54, 58]. Supporting observations of anti-apoptosis due to exercise, treadmill training in a similar rodent model is marked by an attenuation in Bax/Bcl-2 ratios in unstressed hearts [104]. The exact mechanisms responsible for protection in aged exercised hearts are currently unknown. A possible mechanism linked to both exercise and IR injury protection is PKCε. In sedentary hearts from aged animals PKCε levels are quite low, though just a few sessions of moderate intensity treadmill exercise elicit a 3-fold increase in cardiac PKCε content. Another possibility is that HSP72, down-regulated with age and largely unresponsive to IPC stimuli in older hearts, is over expressed by exercise [105]. Given that HSP72 is not essential in the young exercised heart, however, there is reason to question whether HSP72 over expression and IR injury protection are interrelated in the aged exercised heart. Future work is needed to demonstrate whether PKCε and other potentially protective mechanisms precondition the aged exercised heart. 

Despite the unknowns, exercise preconditioning is fundamentally unique from IPC and pharmacologic stimuli in that non-inflammatory pathways mediate the observed protection. This fact may have profound implications for cardiac preconditioning research aimed at discovering viable therapeutic interventions against IR injury. While IPC and pharmacologic preconditioning mediates a short window of cellular protection via COX-2 and iNOS, inflammatory means of cytoprotection are not biologically intended as a long term solution for tissue viability [106]. The rationale presented currently may partially explain anthologized statements from a 2004 position paper by pre-eminent cardiac preconditioning scientists that question why decades of IPC research have not yielded clinical therapies against IR injury [4]. This understanding underpins the need for additional exercise preconditioning research to uncover sustainable mechanisms of cardioprotection in the development of translational cures against IR injury.

### Post Translational Modifications as a Novel Avenue of Cardioprotection Research

Recent research findings suggest that posttranslational protein modifications may be essential in mediating cardioprotection against IR injury. Phosphorylation, methylation, and O-linked β–N-acetyl-glucosamine (O-GlcNAc), events are prime examples of biochemical processes whereby post translational modification may benefit hearts exposed to IR. Phosphorylation of Akt may activate cell survival pathways via PI3K resulting in K_ATP_ channel opening and HSP up-regulation [107, 108]. Redundancy being ever present, iNOS and COX-2 and possibly other down-stream mediators may elicit cardioprotection via JAK-STAT3 phosphorylation [109, 110]. O-GlcNAc derived modifications induce a multitude of cellular responses including signal transduction, metabolic alterations, proteolytic modifications, and anti-apoptotic effects [111]. In the context of IR, O-GlcNAc modifications attenuate IR injury [112]. Methylation of amino acid residues also prevents IR injury, presumably through modification of myocardial homocysteine [113]. Given links between these biochemical events, which are fundamentally linked to ROS production and Ca^2+^ overload, there is reason to suspect that exercise induced cardioprotection may also be mediated by these or similar post translational modifications. To date however direct evidence linking post translational modifications to IR injury prevention is lacking and represents an important avenue for future research. Given that many of the mechanisms linked to post translational modifications, including HSP72, iNOS, and COX-2 have not been found essential to exercise preconditioning, there is reason to suspect that protective mediators may be unique to the exercise stimulus. Further research should examine whether exercise also prevents potentially pathologic post translational modifications as part of the preconditioning process [111].

## CONCLUSION AND SUMMARY

This review summarized almost two decades of research that confirms exercise is cardioprotective against IR injury. Redundant protective mediators are evident in the exercised heart including heat shock proteins, Ca^2+ ^handling proteins, ATP-sensitive potassium channels, and endogenous antioxidant networks. The interplay of these known mediators, in addition to the discovery of novel protective mediators, will undoubtedly continue to emerge and strengthen our appreciation for the value of exercise as a powerful and multifaceted therapeutic in cardiovascular disease care.

## Figures and Tables

**Table 1.** Mediators of Protection Against IR Injury Exercise, Ischemic, and Pharmacologic Preconditioned Hearts

Protective mediator	Ischemic/pharmacologic Preconditioning	Exercise Preconditioning
iNOS	✓	Not elevated
COX-2	✓	Not elevated
HSPs	✓	Not essential
K_ATP_ channels	Sarc K_ATP_ – trigger, Mito K_ATP_ – mediator	Sarc K_ATP_ & Mito K_ATP_ mediator
Endogenous opioids	Not demonstrated	Mediator
MnSOD	Not demonstrated	✓
SERCA-2A preservation	Not demonstrated	✓

Side-by-side comparison of identified mediators of cardiac preconditioning by exercise, ischemic, and pharmacologic stimuli reveal fundamental differences. Mechanisms responsible for ischemic preconditioning, including cardiac iNOS, COX-2, HSPs, and K_ATP_ channels, have been confirmed pharmacologic interventions targeting these respective mediators. With the exception of K_ATP_ channels, exercise preconditioning against IR injury appears to be mediated by alternative mechanisms including endogenous opioids, MnSOD, and improved calcium handling (SERCA-2A preservation). Inducible nitric oxide synthase (iNOS), cyclooxygenase-2 (COX-2), heat shock proteins (HSPs), ATP-sensitive potassium channels (K_ATP_), mitochondrial and sarcolemmal K_ATP_ channels (mito-K_ATP_ and sarc-K_ATP_, respectively), manganese superoxide dismutase (MnSOD), sarco/endoplasmic reticulum calcium ATPase-2A (SERCA-2A).
